# Dendrimers in Layer-by-Layer Assemblies: Synthesis and Applications

**DOI:** 10.3390/molecules18078440

**Published:** 2013-07-17

**Authors:** Katsuhiko Sato, Jun-ichi Anzai

**Affiliations:** Graduate School of Pharmaceutical Sciences, Tohoku University, Aramaki, Aoba-ku, Sendai 980-8578, Japan; E-Mail: satok@m.tohoku.ac.jp

**Keywords:** dendrimer, layer-by-layer film, multilayer film, microcapsule, biosensor, drug delivery, imaging

## Abstract

We review the synthesis of dendrimer-containing layer-by-layer (LbL) assemblies and their applications, including biosensing, controlled drug release, and bio-imaging. Dendrimers can be built into LbL films and microcapsules by alternating deposition of dendrimers and counter polymers on the surface of flat substrates and colloidal microparticles through electrostatic bonding, hydrogen bonding, covalent bonding, and biological affinity. Dendrimer-containing LbL assemblies have been used to construct biosensors, in which electron transfer mediators and metal nanoparticles are often coupled with dendrimers. Enzymes have been successfully immobilized on the surface of electrochemical and optical transducers by forming enzyme/dendrimer LbL multilayers. In this way, high-performance enzyme sensors are fabricated. In addition, dendrimer LbL films and microcapsules are useful for constructing drug delivery systems because dendrimers bind drugs to form inclusion complexes or the dendrimer surface is covalently modified with drugs. Magnetic resonance imaging of cancer cells by iron oxide nanoparticles coated with dendrimer LbL film is also discussed.

## 1. Introduction

**Figure 1:**
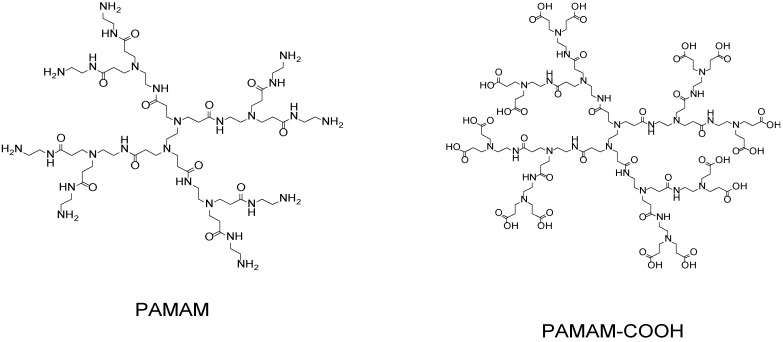
Chemical structures of PAMAM (generation 1) and PAMAM–COOH (generation 1.5) dendrimers.

Dendrimers are a class of synthetic polymers that have monodisperse molecular weight and a well-defined three-dimensional structure consisting of highly branched backbones [1,2,3,4,5], in clear contrast to the linear backbones of conventional synthetic polymers, whose molecular weight is usually dispersed. The size and shape of dendrimers depend on their number of branched units (*i.e.*, generation) and their backbone constituents. Poly(amidoamine) (PAMAM) dendrimers (Figure 1), for example, exhibit a flexible conformation in generation 4 or lower, whereas higher-generation PAMAM dendrimers assume a rigid globular conformation [3]. In general, the size and rigidity of dendrimers increase with increasing number of generations. Dendrimers accommodate small molecules and nanoparticles in their interior through electrostatic or hydrophobic interactions to form host-guest complexes [6,7]. The surface of dendrimers also binds guest molecules through electrostatic binding because polar functionalities, such as amine and carboxyl groups, are located at the dendrimers periphery. In some cases, surface groups are covalently modified with additional functionalities, such as sugars and drugs [8,9]. Therefore, dendrimers are often used as carriers for drugs to deliver them to desired sites. A number of recent reviews cover the structure, binding properties, and biomedical applications of dendrimers [10,11,12,13].

Dendrimers have been actively studied for medical and biotechnology applications, owing to their acceptable biocompatibility. The development of dendrimer-based drug delivery systems is one of the active areas in biomedical applications of dendrimers. For this purpose, a variety of drugs have been encapsulated in the interior of dendrimers [14,15]. In addition, functional groups on the dendrimer surface can be covalently modified with drugs such as doxorubicin, methotrexate, and paclitaxel [9,16,17]. The importance of the chemical nature of linkers for the covalent and non–covalent attachment of drugs to the dendrimer surface has recently been discussed [18]. Gene delivery is another research target, in which positively charged dendrimers are successfully used as vectors for DNA transfection [19]. Magnetic resonance–based and fluorescence-based bio-imaging methods have also been actively studied using magnetically and fluorescently active dendrimers for diagnostic analysis [20,21]. In addition, dendrimers are active components in the development of electrochemical biosensors, in which dendrimers are covalently or non-covalently coupled with electron transfer mediators and confined on the surface of an electrode [22,23]. The high compatibility of dendrimers with proteins, such as enzymes, is an advantage in constructing biosensors. Thus, dendrimers are useful materials for biotechnology applications because desired functionalities can be added through covalent or non-covalent modification.

Another route to enhancing dendrimer functionality is to construct spatially ordered assemblies of dendrimers. In fact, dendrimers have been built into assemblies such as monolayers, multilayers, micelles, vesicles, and microcapsules to construct advanced materials for biomedical and biotechnology applications. Modified and unmodified dendrimers have been used for constructing these two- or three-dimensional assemblies. In recent reports, the construction of dendrimer assemblies and their applications have been reviewed [10,24,25].

Among dendrimer assemblies, multilayered assemblies that are constructed through the layer-by-layer (LbL) deposition of dendrimers have recently attracted much attention because of their facile preparation and versatility in terms of structure and function. The LbL deposition technique was developed by Decher and co-workers in the early 1990s for preparing multilayered thin films with nanometer thickness [26,27]. Since then, LbL films have been widely studied in materials science and technology. Thin films have been prepared by the alternating deposition of positively and negatively charged polymers on the surface of a solid substrate through electrostatic attraction. An advantage of the LbL deposition technique is its wide selection of materials as film components, including synthetic polymers [28,29], oligosaccharides and polysaccharides [30,31,32], DNA [33], and proteins [34,35,36]. The driving force for film deposition is not limited to electrostatic force of attraction; other binding interactions such as charge-transfer interaction [37], cation-dipole interaction [38], hydrogen bonding [39], covalent bonding [40], host-guest complexation [41,42] and biological affinity [43,44,45] are also available depending on the film components. LbL films can be deposited on the surface of any type of material irrespective of surface morphology. A characteristic feature of LbL films is that their thickness strongly depends on the number of depositions or the number of deposited layers. In addition, operational variables in film preparation, such as the pH and ionic strength of the bath solution, the concentration of materials in solution, and deposition time, are crucial factors determining the thickness of the films. In other words, the thickness of LbL films can be controlled on the nanometer scale by adjusting these parameters. It is noteworthy that the chemical and biological activities of proteins and other functional molecules can be preserved in LbL films, suggesting the potential use of these films in biotechnology fields. The synthesis and applications of LbL films have been comprehensively reviewed by many authors [46,47,48,49,50,51,52,53,54,55]. Therefore, this review focuses on the development of LbL assemblies composed of dendrimers and their applications, including bio-sensing, controlled drug release, and bio-imaging. In the following section, we begin with an overview of the synthesis of dendrimer-containing LbL assemblies.

## 2. Synthesis of dendrimer LbL assemblies

### 2.1. Electrostatic Bonding LbL Films

**Figure 2:**
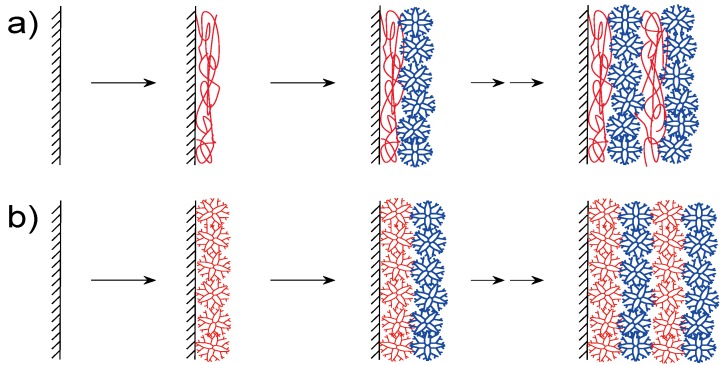
Preparation of (**a**) dendrimer/polymer and (**b**) dendrimer/dendrimer LbL films.

Dendrimers can be built into LbL multilayer films through electrostatic bonding because dendrimers often contain charged surface groups such as amine and carboxyl residues. Thus, LbL films are constructed using positively charged dendrimers and polyanions, or negatively charged dendrimers combined with polycations (Figure 2a). In addition, oppositely charged dendrimers may be employed for constructing dendrimer LbL films without other polymers (Figure 2b). In constructing electrostatic bonding LbL films, one should take into consideration that dendrimers often contain charged tertiary amine groups in their interior as well as primary amines at their periphery [56].

**Figure 3:**
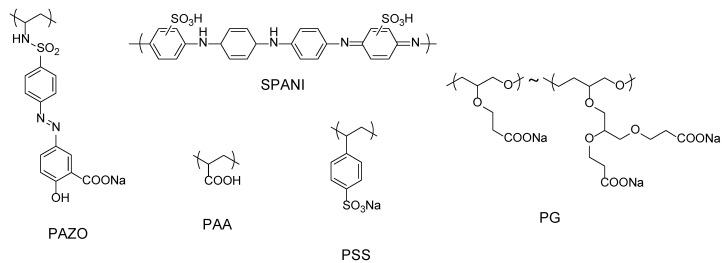
Chemical structures of polyanions used for the construction of dendrimer LbL films.

**Figure 4:**
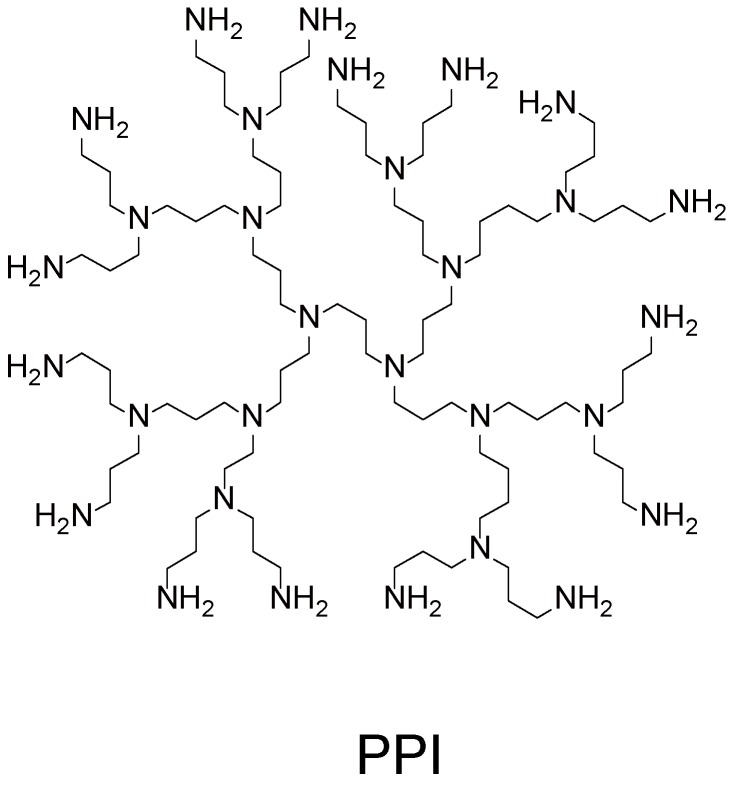
Chemical structure of poly(propyleneimine) (PPI) dendrimer.

Early works demonstrated that all the routes mentioned above can be used in the preparation of dendrimer LbL films. In fact, LbL films were successfully prepared through electrostatic interactions between PAMAM dendrimers with amine and carboxyl surface groups [57]. Oppositely charged PAMAM dendrimers were alternately deposited up to 20 layers on a silicon wafer to form LbL films, in which PAMAM dendrimers were found to be slightly deformed and flattened. LbL films comprising dendrimer and synthetic polymers have also been prepared using different types of polymeric materials, including an azobenzene polymer [58], poly(acrylic acid) (PAA) [59], sulfonated poly(aniline) [60], poly(styrene sulfonate) (PSS) [61] and poly(glycerol) [62]. Figure 3 illustrates the chemical structures of the polymers. In these examples, PAMAM and poly(propylenimine) (PPI) dendrimers (Figure 4) with positive surface charges were built into LbL architectures through electrostatic interactions with polyanions. Interestingly, the polymers deposited on the outermost surface of LbL film often desorbed in part when the next dendrimer layer was deposited (*i.e.*, adsorption-desorption behavior), depending on the pH and ionic strength of the solutions [58,60,61]. Kim and Bruening observed the pH–dependent growth of PAMAM/PAA films. They obtained the maximum film thickness when the film was deposited using PAMAM solution at pH 8 and PAA solution at pH 4, at which both PAMAM and PAA are only partially charged. The bilayer thickness of the films can be tuned from 1 to 400 nm by varying deposition pH [59]. LbL films composed of Au or Ag particle-encapsulating PAMAM dendrimers have also been constructed through electrostatic bonding [63,64].

### 2.2. Hydrogen-Bonding LbL Films

**Figure 5:**
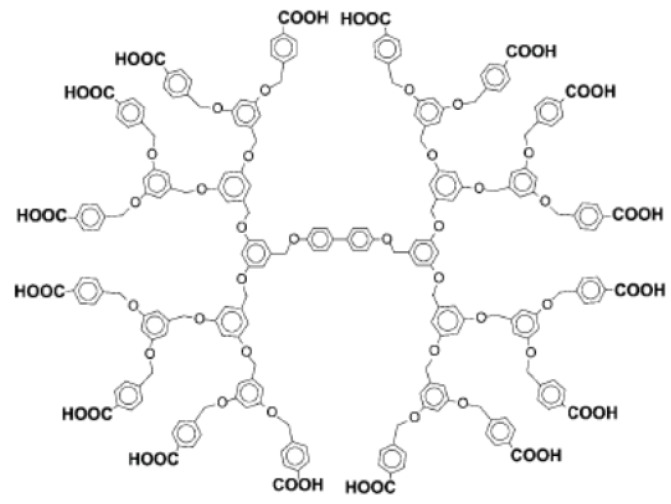
Chemical structure of carboxyl-terminated dendrimer (DEN-COOH). Reprinted with permission from Zhang *et al.* [65] Copyright (2012) The Royal Society of Chemistry.

Hydrogen bonding is another driving force for the formation of LbL films [39]. In this context, dendrimers with carboxylic acid residues on the periphery are used as film components because carboxylic residues can serve as hydrogen-bonding donors and acceptors. Zhang’s group prepared single-component LbL films by using a carboxylic acid-terminated dendrimer (DEN-COOH, Figure 5) as the hydrogen bonding donor and acceptor [65]. Two-component LbL films were also prepared by the alternating deposition of DEN-COOH as the hydrogen bonding donor and poly(4-vinylpyridine) (PVP) as the hydrogen bonding acceptor [66,67]. Carboxyl-terminated PAMAM dendrimers (PAMAM-COOH, Figure 1) were also employed as building blocks of LbL films, in which PAMAM-COOH was combined with poly(carboxylic acid)s, such as poly(methacrylic acid) (PMA) and PAA. PAMAM-COOH/PMA films were prepared at pH 4.0 through hydrogen bonding between carboxylic acid residues in PAMAM-COOH and PMA; however, the LbL films decomposed at neutral pH as a result of breakage of the hydrogen bonds owing to the deprotonation of carboxylic acid residues [68,69]. The pH stability of the LbL films depended on the acidity of the counter polymer [70]. The researchers suggested a potential use of PAMAM-COOH-based LbL films as stimulus-sensitive devices. Ito and Imae suggested that PAMAM-COOH forms monolayers and multilayers on the surface of metal substrates [71].

### 2.3. Covalent-Bonding LbL Films

**Figure 6:**
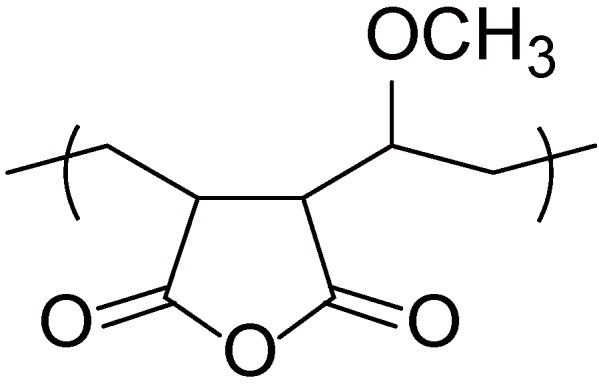
Chemical structure of poly(maleic anhydride)-*co*-poly(methyl vinyl ether).

LbL films with covalent linkages between layers should be more stable than those prepared through electrostatic affinity and hydrogen bonding. Covalent bonding LbL films were constructed through the alternating deposition of PAMAM or PPI dendrimer and a reactive polymer, poly(maleic anhydride)-*co*-poly(methylvinyl ether) (Figure 6) [72,73]. The anhydride group in the polymer reacted with primary amines on the dendrimer surface to form amide and imide linkages in the LbL films. Another group also reported a protocol for constructing covalent-bonding LbL films of dendrimers based on Schiff’s base formation, in which amine-terminated PAMAM and a peroxidate (IO_4_^−^)-oxidized enzyme were used [74,75]. Photochemical reaction was employed to introduce covalent bonding in LbL films consisting of PAMAM-COOH and diazo resin [76,77,78].

### 2.4. Miscellaneous

Watanabe and Regen constructed dendrimer LbL films based on coordination chemistry, where a solid substrate was alternately immersed in solutions of PAMAM dendrimer and K_2_PtCl_4_ in dimethylsulfoxide [79]. They found a linear increase in film thickness up to 12–16 bilayers. Anzai and co-workers established a protocol for preparing dendrimer LbL films through the biological affinity between avidin and biotin [80,81]. Avidin is a tetramer protein that contains four identical sites for binding to biotin [82]. Therefore, polymeric materials labeled with multiple biotin residues can be adsorbed to an avidin-modified surface through the avidin-biotin affinity, leaving free biotin residues for further binding of the next avidin layer [83,84,85,86]. Indeed, the alternating deposition of avidin and a biotin–labeled PAMAM dendrimer gave LbL films, in which the PAMAM dendrimer was found to provide nearly monomolecular coverage in each layer [81].

## 3. Applications of Dendrimer LbL Assemblies

### 3.1. Biosensors

Biosensors are analytical tools that can be used for determining ions and molecules in biological fluids. They are often fabricated using electrodes or optical transducers coupled with catalytic or molecular recognition elements such as enzymes and antibodies. Consequently, immobilizing proteins on the surface of transducers without loss of their biological activity is a key issue in the construction of biosensors. For this purpose, protein-containing LbL films have been widely studied to improve the performance of biosensors [87,88,89,90,91].

In this context, the significant roles of dendrimers in biosensor construction can be envisaged, including: (1) forming surface monolayers as scaffolds for protein immobilization; (2) depositing LbL multilayer films composed of dendrimers and proteins; and (3) encapsulation or covalent binding of metal particles and electron transfer mediators. Several groups have used dendrimers to modify the surface of electrodes and other devices with monolayer films, on which proteins are immobilized covalently or non-covalently. Monomolecular layers of PAMAM dendrimer were prepared on the surface of Au or Ag substrates for surface plasmon sensors [92,93,94]. PAMAM monolayers were further modified with proteins or single–stranded DNA (ssDNA) to fabricate biosensors. In these examples, primary amines in PAMAM dendrimer were covalently coupled with the substrate surface and biomolecules. Other groups reported electrochemical biosensors prepared using electrodes modified with a dendrimer monolayer coupled with an aptamer and Pt nanoclusters [95,96]. The well-defined and compact conformation of dendrimers is beneficial in designing biosensor interfaces.

Several groups studied the preparation of dendrimer/enzyme multilayer films and their applications in biosensors. LbL films composed of PAMAM dendrimer and glucose oxidase (GOx) were coated on the surface of Au electrodes to construct glucose biosensors, in which PAMAM layers were covalently coupled with GOx through Schiff’s base linkages [74]. The output current of the glucose sensors linearly increased with increasing number of PAMAM/GOx bilayers up to 5, confirming that the catalytic activity of GOx was preserved in the LbL film. These results suggest that glucose can smoothly permeate porous PAMAM/GOx multilayer films. In a similar protocol, ferrocene–tethered PAMAM was also used for constructing reagent-free glucose sensors that can be used without adding an electron transfer mediator to the sample solution [75]. Another group showed the potential use of LbL films consisting of ferrocene–tethered PAMAM and Au nanoparticles for constructing amperometric biosensors [97]. PAMAM/GOx LbL films were prepared through electrostatic binding between an amine–terminated PAMAM dendrimer and negatively charged GOx at neutral pH [98]. Only two bilayer films, (PAMAM/GOx)_2_, can be prepared; however; further deposition of GOx layers did not enhance the output current of glucose sensors. Thus, covalent bonding PAMAM/GOx films are superior to electrostatic bonding films for constructing glucose biosensors. The compact globular conformation of PAMAM may be less effective in forming complementary electrostatic bonds to GOx, which is in clear contrast to the successful binding of linear poly(amine)s [99] and lectin [100] to GOx. On the other hand, LbL films composed of Cl-catechol-1,2-dioxigenase and PAMAM dendrimer were stable even though they were constructed through electrostatic bonding [101]. Hu and co-workers studied the construction of electrostatic bonding LbL films of PPI dendrimer and heme proteins under different pH conditions [102]. Interestingly, hemoglobin (Hb)/PPI multilayer films could be successfully assembled at pH 9.0, at which Hb is negatively charged, as well as at pH 5.0, at which both Hb and PPI are positively charged. The LbL film formation at pH 5.0 was ascribed to the localized electrostatic interactions or charge reversal of Hb induced on the PPI surface. These results suggest that the stability of electrostatic bonding dendrimer/protein films significantly depends on the type of protein, probably due to different charge distributions on the surface of proteins.

Metal nanoparticles are successfully encapsulated into dendrimers to form protected metal nanoparticles [7]. Thus, Au and Pt nanoparticle-encapsulating dendrimers have been widely used in constructing enzyme LbL films for biosensor applications. Hu and Zhang prepared LbL films consisting of Au nanoparticle-encapsulating PPI dendrimer and myoglobin on the surface of graphite electrodes. The catalytic response of the electrodes coated with Au-PPI dendrimer films was higher than that of the electrodes modified with LbL films without Au nanoparticles [103]. PAMAM dendrimers encapsulating CdS semiconductor or Pt were also used for constructing glucose biosensors [104,105]. In other work, Pt^−^ or Au^−^encapsulating dendrimers were coupled with carbon nanotubes and enzymes in LbL films for constructing biosensors sensitive to pesticides [106] and glutamate [107,108]. The modification of dendrimer LbL films with metal hexacyanoferrate nanoparticles was effective for enhancing the electrochemical response of biosensors to hydrogen peroxide [109,110] and glucose [111].

**Figure 7:**
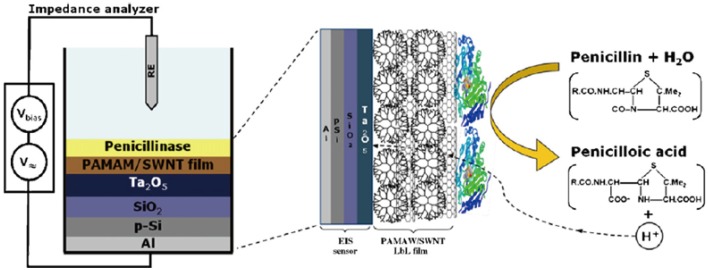
Penicillin FET biosensor fabricated based on dendrimer LbL film and penicillinase. Reprinted with permission from Schöning *et al.* [112] Copyright (2009) Elsevier.

Recently, dendrimer LbL films have been employed as gate materials for field effect transistor (FET) biosensors. Schöning and co-workers prepared LbL films by alternating deposition of carboxylated single-walled carbon nanotubes and PAMAM dendrimer on the surface of an FET gate to fabricate penicillin biosensors (Figure 7) [112,113]. The gate potential of the FET biosensor was sensitive to penicillin in the concentration range of 5.0 × 10^−6^ to 2.5 × 10^−2^ M. Zucolotto and co-workers used LbL films composed of tetrasulfonated phthalocyanine (TsPc) and dendrimers to construct FET sensors sensitive to pH [114], humidity [115], and glucose [116]. These authors ascribed the high performance of the FET sensors to the porous structure of the TsPc/dendrimer LbL films permeable to H^+^ and glucose.

### 3.2. Encapsulation and Controlled Release

As briefly discussed in the introduction section, dendrimers have been used as vehicles for drug and gene delivery because they can accommodate these molecules in their interior and on their surface [14,15,16,17,18,19]. LbL-deposited thin films and microcapsules have also been studied as scaffolds or microcontainers for controlled drug release [55,117,118,119,120]. In this context, dendrimer-containing LbL films would have promising applications in drug delivery and related systems. Dendrimer-containing LbL films are expected to provide multiple binding sites to drugs in the dendrimer interior, on the dendrimer surface, and in the entire film.

LbL assemblies comprising PAMAM dendrimer and PSS were deposited on the surface of a flat substrate and on poly(styrene) microbeads to study the loading and release of a model dye, carboxylated fluorescein (CF) [121]. The model dye was trapped in PAMAM/PSS films upon exposing the films to CF solution. Tertiary and primary amine residues of PAMAM most likely provide binding sites to negatively charged CF. The bound CF was released from the LbL films into 0.154 M NaCl solution at pH 6.5 according to Fickian-type kinetics. The release rate was rather high; above 75% of bound CF was released in the first 60 min. Other researchers reported the construction of dendrimer/liposome LbL films for the loading and release of ibuprofen [122,123]. Stimulus-sensitive materials are crucial in the development of drug delivery systems. Toward this goal, LbL films sensitive to pH [118], salts [124], temperature [125], sugars [126], and electric signals [127] have been studied. Hydrogen-bonding LbL films are typical example of pH-sensitive films because poly(carboxylic acid), a typical component of LbL films, dissociates at neutral/basic pH, resulting in the breakage of hydrogen bonds. Using this strategy, Zhang and co-workers demonstrated the pH-induced release of film components from PAMAM-COOH/PVP multilayer films [128]. PAMAM-COOH/PVP films were stable at acidic and neutral pH but decomposed at pH 12 and 13. PAMAM-COOH/PMA and PAMAM-COOH/PAA films were more sensitive to pH changes; these films decomposed at pH 5.5 and 5.0 or higher, respectively [69,70]. Model dyes, such as Rose Bengal and sulfonated tetraphenylporphyrin, were loaded and released from PAMAM-COOH/PMA films in response to pH changes [69].

**Figure 8:**
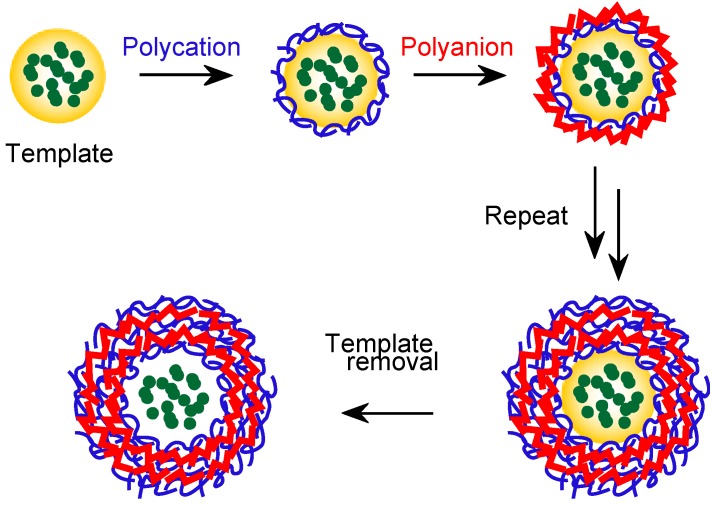
Construction of LbL film–based microcapsules. Reprinted (adapted) with permission from Sato *et al*. [120] Copyright (2011) The Japan Society for Analytical Sciences.

Recently, much attention has been devoted to LbL microcapsules, which are prepared through the alternating deposition of polymers on the surface of colloid particles as the template followed by the dissolution of template materials (Figure 8) [55,117,118,119,120]. LbL microcapsules with dendrimer shells are interesting because such microcapsules provide two distinct binding sites in the capsule interior and on the capsule shell. PAMAM/PSS film-based microcapsules were prepared by Khopade and Caruso to study the loading and release of the anticancer drug doxorubicin (DOX) [129]. The release of DOX from the microcapsules was sustained for several hours in 0.154 M NaCl solution. The use of Au nanoparticle-encapsulating PAMAM enhanced the stability of PAMAM/PSS microcapsules [130]. LbL microcapsules with phosphorus dendrimers coupled with linear polymers or DNA were studied in relation to their mechanical properties. The phosphorus dendrimer microcapsules were found to be softer than microcapsules assembled from linear polyelectrolytes; however, the microcapsules were stiffened by the treatment with organic solvent [131,132,133].

**Figure 9:**
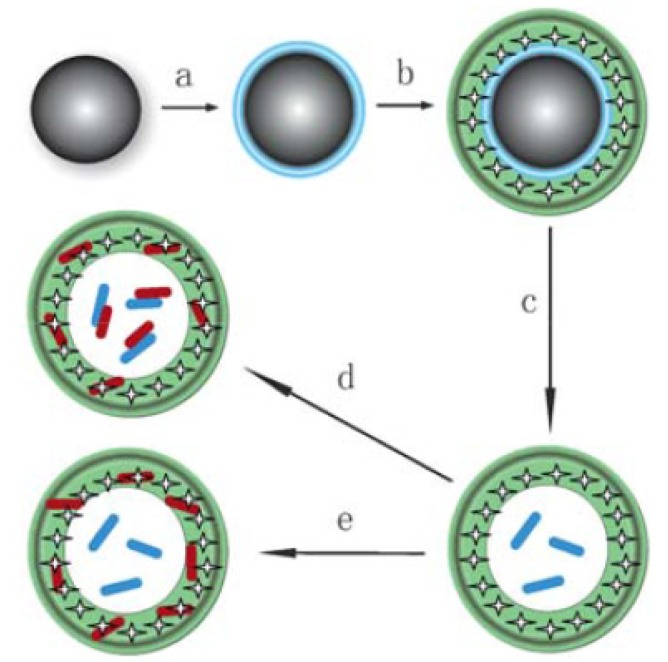
Preparation of ssDNA-containing LbL microcapsule and selective encapsulation of target ssDNA into the microcapsule through hybridization. (**a**) deposition of ssDNA on the core particle; (**b**) deposition of dendrimer film; (**c**) removal of core material; (**d**) encapsulation of target ssDNA into the microcapsule; and (**e**) adsorption of mismatch ssDNA. Reprinted with permission from Zhang *et al.* [135] Copyright (2010) The Royal Society of Chemistry.

For drug delivery using microcapsules, a prerequisite is to regulate the amount of drug loading. However, precise control of drug loading in microcapsules is rather difficult because drugs are loaded simply by dispersing hollow microcapsules in drug solutions, where drug molecules are loaded according to the concentration gradient. Recently, a protocol for the selective encapsulation of an ssDNA into microcapsules with a phosphorus dendrimer/PSS shell has been reported by Feng and co-workers [134,135]. Microcapsules containing probe ssDNA can be loaded with a target ssDNA through DNA hybridization by dispersing the microcapsules in the target solution; however, mismatch ssDNA is not encapsulated (Figure 9). The addition of detergent, such as sodium dodecyl sulfate, in the double–stranded DNA (dsDNA)-loaded microcapsule solution may induce the dehybridization of the dsDNA, resulting in the release of ssDNA. Thus, biological interactions effectively improve the loading selectivity of microcapsules. Protein-loaded microcapsules may also be useful for the selective and controlled encapsulation of biomolecules [136,137]. Silver sulfadiazine (AgSD), an antibiotic with limited water solubility, was coupled with LbL films composed of oppositely charged PAMAM dendrimers to improve aqueous solubility and antibiotic activity [138]. In this formulation, AgSD microparticles were coated directly with the dendrimer LbL films. Consequently, the loading of drug components into the microcapsules was high, in contrast to the loading of drugs into previously prepared microcapsules. Cream formulations containing AgSD nanoparticles coated with PAMAM LbL films exhibited higher antibacterial activity than the formulation without PAMAM coating. Notably, the dendrimer itself possesses antifungal and antimicrobial activities [139,140,141].

Also possible is encapsulating PAMAM dendrimers in microcapsules prepared using poly(allylamine) and poly(vinyl sulfate) as shell materials. PAMAM-containing microcapsules were further loaded with fluorescent 1-anilinonaphthalene-8-sulfonic acid (ANS) as a model drug by binding ANS to the dendrimers. The rate of ANS uptake was determined by the rate of ANS transport across the capsule shell, while the dissociation of ANS from PAMAM was the rate-determining step for ANS release out of the capsule [142].

### 3.3. Miscellaneous

Iron oxide (Fe_3_O_4_) nanoparticles coated with LbL film have been prepared for targeting and imaging cancer cells. The surface of Fe_3_O_4_ nanoparticles was coated with poly(lysine) (PL)/poly(glutamic acid) (PGA) LbL layers followed by cross-linking with carbodiimide reagent, and the outermost surface of the LbL layer was modified with folic acid (FA)-tagged PAMAM [143]. Cross-linking the PL/PGA layers was essential for stabilizing the modified Fe_3_O_4_ nanoparticles [144]. The FA-modified Fe_3_O_4_ nanoparticles can target FA receptors (FARs) that are overexpressed on the surface of cancer cells. The FA-modified Fe_3_O_4_ nanoparticles targeted cancer cells and tumor models at a volume as small as 0.60 ± 0.15 cm^3^ and were successfully used for magnetic resonance imaging of the cells.

The adsorption of cells and proteins on the surface of PAMAM dendrimer LbL films has been studied to evaluate the biocompatibility of the films. PAMAM/PSS LbL films coated on the surface of flat substrates and microparticles were further modified with polyethylene glycol (PEG)-bearing lipids to suppress the adsorption of human serum albumin and the macrophage cell line [145]. The PEG-modified surface of the LbL films was found to be resistant to cell adhesion and HSA adsorption, as compared with the surface of unmodified positively charged PAMAM/PSS films. In another study, the influence of the surface charges of hydrazine phosphorus dendrimer LbL films on the adhesion and maturation of fetal cortical rat neurons was evaluated. The neurons firmly adhered and matured faster on LbL film surfaces with positive charge than on those with negative charges [146].

In this paper, synthesis of dendrimer-containing LbL assemblies and their applications in medical and biotechnology fields have been comprehensively reviewed. It is noteworthy that dendrimer LbL assemblies exhibit characteristic features in the synthesis and applications. For example, adsorption-desorption behavior is often observed upon synthesizing dendrimer LbL films [58,60,61], in contrast to a linear growth of LbL films composed of conventional polymers.

## 4. Conclusions

Dendrimers have been successfully assembled in LbL films and microcapsules through electrostatic bonding, hydrogen bonding, covalent bonding, and biological affinity. An advantage of dendrimers as building blocks of LbL assemblies is the facile preparation of chemically modified dendrimers. The surface reactive groups of dendrimers such as primary amine and carboxylic acid residues can be covalently or non-covalently modified with desired functional groups. It is also possible to accommodate small molecules or nanoparticles in the interior of dendrimers. Consequently, dendrimer-containing LbL films and microcapsules have found a variety of applications in medicine and biotechnology. Dendrimer LbL films have widely been used in the construction of biosensors, in which dendrimers are often modified with electron transfer mediators or metal nanoparticles to enhance biosensor response. The development of drug delivery systems is another promising goal of research on dendrimer-containing LbL films and microcapsules. For this purpose, drugs are attached to the dendrimer surface or included in the dendrimer interior through host-guest complexation. In addition, Fe_3_O_4_ nanoparticles coated with dendrimer LbL film have been used for the targeting and magnetic resonance imaging of cancer cells. Acceptable biocompatibility of modified dendrimers is advantageous in the biological applications of dendrimers. Accordingly, dendrimer-containing LbL assemblies are expected to find further applications in various areas of medicine and biotechnology.
